# Placenta-Derived Mesenchymal Stem Cells (pMSCs) Reverse Diabetes-Associated Endothelial Complications in a Preclinical Animal Model

**DOI:** 10.3390/ijms26168057

**Published:** 2025-08-20

**Authors:** Yasser Basmaeil, Ahmed Bakillah, Abdullah Mohammed Al Subayyil, Haya Nasser Bin Kulayb, Maha Abdullah AlRodayyan, Abeer Al Otaibi, Sindiyan Al Shaikh Mubarak, Hassan S. Alamri, Altaf A. Kondkar, Jahangir Iqbal, Tanvir Khatlani

**Affiliations:** 1 Stem Cell Research Unit, Blood and Cancer Research Department, King Abdullah International Medical Research Center (KAIMRC), King Saud bin Abdulaziz University for Health Sciences (KSAU-HS), Ministry of National Guard Health Affairs (MNGHA), Riyadh 11426, Saudi Arabia; alsubayyila@kaimrc.edu.sa (A.M.A.S.); binkulaybh@kaimrc.edu.sa (H.N.B.K.); alrodayyanm@kaimrc.edu.sa (M.A.A.); khatlanit@kaimrc.edu.sa (T.K.); 2 Biomedical Research Core, King Abdullah International Medical Research Center (KAIMRC), King Saud bin Abdulaziz University for Health Sciences (KSAU-HS), Ministry of National Guard Health Affairs (MNGHA), Al Ahsa 31982, Saudi Arabia; bakillaha@kaimrc.edu.sa (A.B.); otaibiabe@kaimrc.edu.sa (A.A.O.); alshaikhmubaraks@kaimrc.edu.sa (S.A.S.M.); jiqbal30@hotmail.com (J.I.); 3 Department of Clinical Laboratory Sciences, College of Applied Medical Sciences, King Abdullah International Medical Research Center (KAIMRC), King Saud bin Abdulaziz University for Health Sciences (KSAU-HS), Ministry of National Guard Health Affairs (MNGHA), Riyadh 11481, Saudi Arabia; amrih@ksau-hs.edu.sa; 4 Department of Ophthalmology, College of Medicine, King Saud University, Riyadh 12372, Saudi Arabia; akondkar@gmail.com

**Keywords:** placental mesenchymal stem cells (pMSCs), endothelial dysfunction, streptozotocin (STZ), type 1 diabetes, glucose tolerance test (GTT), insulin tolerance test (ITT)

## Abstract

Diabetes is increasingly recognized as a chronic inflammatory disease marked by systemic metabolic disturbances, with endothelial dysfunction playing a central role in its complications. Hyperglycemia, a hallmark of diabetes, drives endothelial damage by inducing excessive reactive oxygen species (ROS) production, particularly hydrogen peroxide (H_2_O_2_). This oxidative stress impairs endothelial cells, which are vital for vascular health, leading to severe complications such as diabetic nephropathy, retinopathy, and coronary artery disease—major causes of morbidity and mortality in diabetic patients. Recent studies have highlighted the therapeutic potential of placenta-derived mesenchymal stem cells (pMSCs), in mitigating these complications. pMSCs exhibit anti-inflammatory, antioxidant, and tissue-repair properties, showing promise in reversing endothelial damage in laboratory settings. To explore their efficacy in a more physiologically relevant context, we used a streptozotocin (STZ)-induced diabetic mouse model, which mimics type 1 diabetes by destroying pancreatic beta cells and causing hyperglycemia. pMSCs were administered via intra-peritoneal injections, and their effects on endothelial injury and tissue damage were assessed. Metabolic tests, including glucose tolerance tests (GTTs) and insulin tolerance tests (ITTs) revealed that pMSCs did not restore metabolic homeostasis or improve glucose regulation. However, histopathological kidney, heart, and eye tissue analyses demonstrated significant protective effects. pMSCs preserved glomerular structure in the kidneys, protected cardiac blood vessels, and maintained retinal integrity, suggesting their potential to address diabetes-related tissue injuries. Although these findings underscore the therapeutic potential of pMSCs for diabetic complications, further research is needed to optimize dosing, elucidate molecular mechanisms, and evaluate long-term safety and efficacy. Combining pMSCs with other therapies may enhance their benefits, paving the way for future clinical applications.

## 1. Introduction

Despite remarkable advancements in medical science, diabetes, particularly type 1 diabetes (T1D), continues to pose significant health challenges. Individuals with T1D are highly susceptible to a variety of complications, with vascular disorders being among the most severe and life-threatening. These complications are closely linked to increased mortality rates and are driven by progressive structural and functional abnormalities in both the microvasculature and microvasculature. Over time, these abnormalities lead to devastating clinical outcomes such as nephropathy (kidney disease), retinopathy (eye disease), neuropathy (nerve damage), and cardiovascular diseases including coronary artery disease [1,2,3].

A central player in the development of these complications is endothelial dysfunction, which is a hallmark of diabetes. The endothelium, a thin layer of cells lining blood vessels, plays a critical role in maintaining vascular homeostasis by regulating blood flow, inflammation, and coagulation. In diabetes, chronic hyperglycemia (elevated blood glucose levels) and oxidative stress disrupt endothelial function, leading to vascular injury [4]. Elevated glucose levels trigger the overproduction of reactive oxygen species (ROS), such as hydrogen peroxide (H_2_O_2_), within endothelial cells. ROS are highly reactive molecules that cause oxidative damage to cellular components, including lipids, proteins, and DNA. This oxidative stress impairs the endothelial lining, reducing its ability to produce nitric oxide (NO), a key molecule that promotes vasodilation and maintains vascular health. As a result, the blood vessels become more prone to inflammation, thrombosis, and atherosclerosis, contributing to the progression of diabetic complications [5,6,7].

Several biomarkers have been identified to assess endothelial dysfunction in T1D. These include elevated levels of von Willebrand Factor (vWF), a protein involved in blood clotting; thrombomodulin, a glycoprotein that regulates coagulation; selectins, which mediate leukocyte adhesion to the endothelium; plasminogen activator inhibitor-1 (PAI-1), which inhibits fibrinolysis (the breakdown of blood clots); type IV collagen, a component of the basement membrane; and tissue plasminogen activator (t-PA), which promotes clot dissolution. These biomarkers reflect the ongoing vascular damage and inflammation in diabetic patients and provide insights into the severity of endothelial dysfunction [8,9,10,11].

In recent years, stem cell biology has emerged as a groundbreaking field in therapeutic and regenerative medicine. Stem cells, with their unique ability to self-renew and differentiate into various cell types, offer immense potential for treating a wide range of diseases, including cardiovascular diseases, immunological disorders, cancer, diabetes, and injuries to organs such as the liver, heart, and brain [12]. Among the various types of stem cells, mesenchymal stem cells (MSCs) have garnered significant attention due to their versatility and therapeutic properties. MSCs are multipotent cells that can be isolated from adult tissues such as adipose tissue, bone marrow, umbilical cord, dental pulp, and the placenta [13]. They exhibit key characteristics, including self-renewal, differentiation potential, low immunogenicity, and the ability to form colonies on plastic surfaces. MSCs can differentiate into various cell types, such as chondrocytes (cartilage cells), adipocytes (fat cells), and astrocytes (glial cells in the brain). Their unique biological properties, such as their ability to migrate to sites of injury or inflammation and modulate immune responses, make them attractive candidates for treating immunological and metabolic disorders, including diabetes and cancer [14].

In previous studies, we isolated and characterized MSCs from different regions of the human term placenta, including decidua basalis MSCs (DBMSCs), decidua parietalis MSCs (DPMSCs), and chorionic villus MSCs (CVMSCs), collectively referred to as placenta-derived MSCs (pMSCs). These cells demonstrated the capacity to differentiate into mesenchymal lineages and secrete a variety of cytokines, growth factors, and immunomodulatory molecules [15]. pMSCs have been shown to protect endothelial cells from inflammatory activation, such as monocyte adhesion while promoting endothelial cell proliferation [16]. Additionally, they exhibit protective effects against endothelial dysfunction caused by ROS, particularly H_2_O_2_-induced oxidative stress [17]. These findings highlight the therapeutic potential of pMSCs in mitigating endothelial dysfunction, making them a compelling candidate for further investigation in animal models of diabetes-induced vascular complications.

The aim of the study is to evaluate the efficacy and feasibility of pMSCs in treating diabetic hyperglycemia and diabetes-induced endothelial dysfunction by administering pMSCs intraperitoneally to streptozotocin (STZ)-induced diabetic mice. STZ is a chemical that selectively destroys pancreatic beta cells, mimicking the pathophysiology of T1D. Serum and histological recovery was assessed using metabolic and histochemistry assays.

## 2. Results

### 2.1. Validation of Diabetes Induction in STZ-Injected Mouse Model

To confirm diabetes induction in the STZ-injected mouse model, blood serum was analyzed for changes in glucose and lipid profiles before DBMSC administration. Blood samples were collected on day 0 (baseline) and day 28 post-STZ injection. Plasma was separated and assessed for glucose, triglycerides, phospholipids, cholesterol, and free fatty acids. The STZ-injected diabetic group exhibited a significant increase in plasma glucose levels compared to baseline (Supplementary Figure S2A). Plasma triglycerides decreased significantly post-STZ injection (Supplementary Figure S2B), while phospholipids increased significantly (Supplementary Figure S2C). Conversely, plasma cholesterol and free fatty acids showed significant decreases in the diabetic group compared to baseline (Supplementary Figure S2D,E). pMSC intervention did not alter these parameters in either the untreated or STZ-induced diabetic groups, as shown in Supplementary Figure S2A–D. These results confirm successful diabetes induction in the STZ mouse model, which is characterized by hyperglycemia and altered lipid profiles, thereby providing a validated model for evaluating therapeutic effects of pMSC.

### 2.2. pMSC Injection Does Not Alter Glucose Homeostasis in STZ-Induced Diabetic Mice

**Figure 1:**
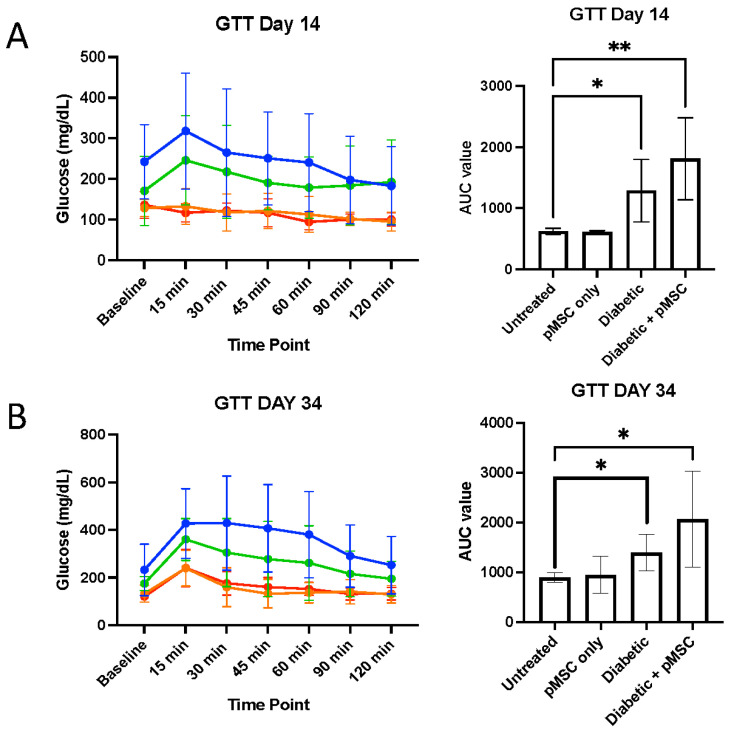
**Glucose tolerance test (GTT) Analysis:** Diabetic mice were administered placental mesenchymal stem cells (pMSCs). On day 14 (**A**) and day 34 (**B**) post-streptozotocin (STZ) injection, diabetic mice treated with pMSCs exhibited a significant lack of response to the transplantation and failed to achieve normoglycemia. Line colors: red = untreated control; orange = pMSC-only treatment; green = diabetic control; blue = diabetic + pMSC treatment. Results are presented as the mean ± standard deviation of the mean (SD) (n = 6 per group; * *p* < 0.05, ** *p* < 0.01 indicates significant differences).

**Figure 2:**
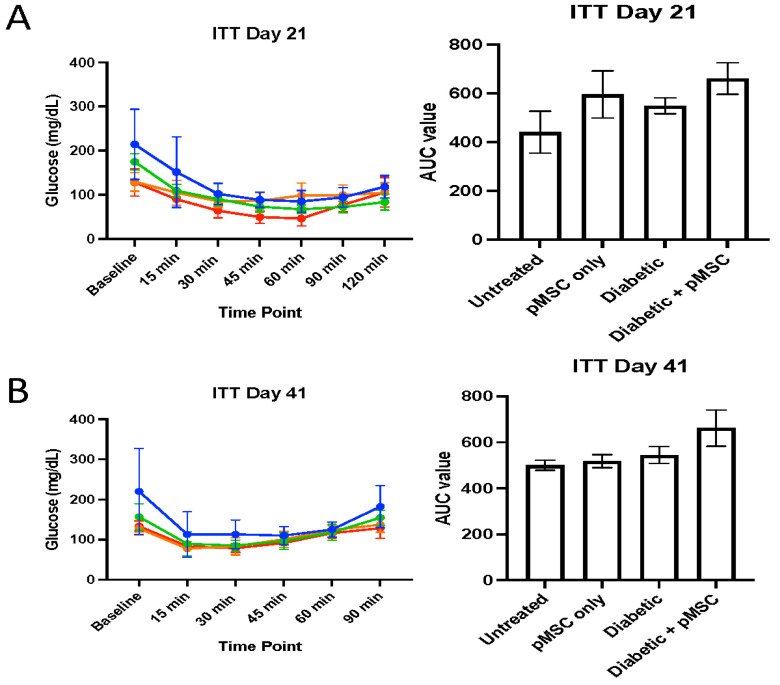
**Insulin tolerance test (ITT) Analysis:** On day 21 (**A**) and day 41 (**B**) post-STZ injection, diabetic mice treated with pMSCs showed no response to the transplantation and failed to achieve normoglycemia. Line colors: red = untreated control; orange = pMSC-only treatment; green = diabetic control; blue = diabetic + pMSC treatment. Results are presented as the mean ± standard deviation of the mean (SD) (n = 6 per group).

To evaluate the impact of pMSCs on glucose metabolism in STZ-induced diabetic mice and untreated controls, we conducted glucose tolerance tests (GTTs) and insulin tolerance tests (ITTs) in STZ-induced diabetic mice and untreated controls. GTT was performed on days 14 and 34 post-STZ administration, while ITT was conducted on days 21 and 41. For GTT, baseline glucose levels were measured, followed by an intraperitoneal (i.p.) injection of 1 g/kg glucose. Blood glucose levels were monitored at 15, 30, 45, 60, 90, and 120 min post-injection. No significant differences were observed in fasting or post-glucose challenge glucose levels between groups (Figure 1A). However, the glucose area under the curve (AUC) for GTT showed a significant difference between the untreated group, diabetic group, and pMSC-treated diabetic group with an AUC Mean Difference of −663 and −1185, respectively (*p*-values < 0.05). No significant differences were observed between other experimental groups (Figure 1B). For ITT, baseline glucose levels were measured, followed by an i.p. injection of 0.75 U insulin/kg. Blood glucose levels were monitored 15, 30, 45, 60, 90, and 120 min post-injection. No significant differences between groups were observed in fasting or post-insulin challenge glucose levels (Figure 2A). Unlike GTT, the glucose AUC for ITT showed no significant difference between the untreated group and the pMSC-treated diabetic group and the other groups (Figure 2B). These results indicate that pMSC injections did not significantly alter glucose homeostasis or improve insulin sensitivity in STZ-induced diabetic mice, suggesting that pMSCs do not directly influence systemic glucose metabolism in this model.

### 2.3. pMSC Administration Reduces Collagen Deposition in the Kidneys of STZ-Induced Diabetic Mice

**Figure 3:**
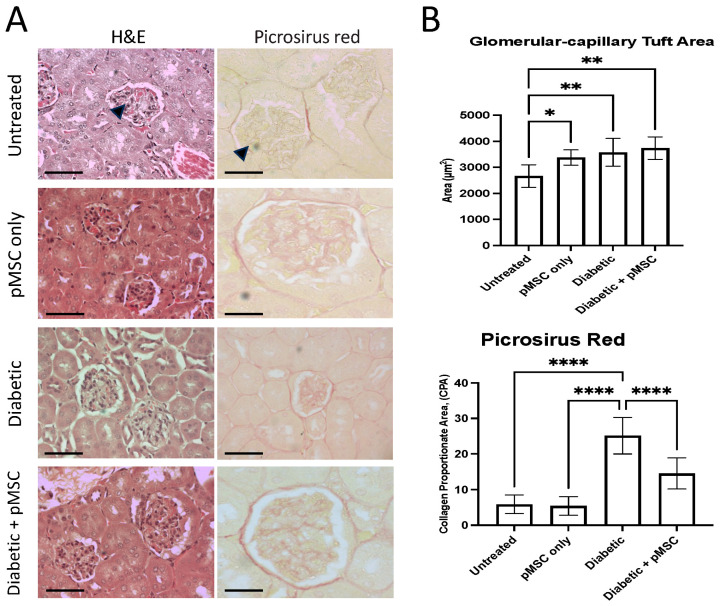
**Histological analysis of kidney tissue:** Representative images and quantitative analysis of hematoxylin and eosin (H&E) staining (**left panel**) and Picrosirius red staining (**right panel**) of kidney sections (**A**). Top panel (**B**): Area for measurement of the glomerular capillary tuft. Quantification of collagen deposition in the glomerulus is represented below panel (**B**). Images of H&E and Picrosirius red-stained sections were acquired at 40X magnification (scale bar = 20 μm); arrows indicate the glomerular capillary tuft area. Bar chart values represent the mean ± standard deviation (n = 4). *, **, **** Indicates significant differences (*p* < 0.05, *p* < 0.01, *p* < 0.0001).

To assess the impact of pMSCs on kidney pathology in diabetic mice, 4 µm thick kidney sections were stained with hematoxylin and eosin (H&E) and imaged using a bright-field microscope (Figure 3A). Image analysis software (ImageJ, version 1.46) was used to measure the glomerular capillary tuft area. A significant increase (*p*-value < 0.05) in glomerular capillary area was observed in the pMSCs-treated, diabetic, and diabetic + pMSCs groups compared to the untreated control group (Figure 3B, top). Kidney sections were stained with Picrosirius red to evaluate collagen deposition and analyzed using ImageJ. Low collagen deposition was observed in both the untreated control group and the pMSC-treated group. However, a significant reduction (*p*-value < 0.05) in collagen deposition was noted in the pMSC-treated diabetic group compared to the untreated diabetic group (Figure 3B, bottom). These findings suggest that pMSC administration mitigates collagen accumulation in the kidneys of STZ-induced diabetic mice, indicating a potential protective effect against diabetic nephropathy. This reduction in collagen deposition may contribute to preserving kidney structure and function in diabetic conditions.

### 2.4. pMSC Administration Restores Retinal Thickness in STZ-Induced Diabetic Mice

**Figure 4:**
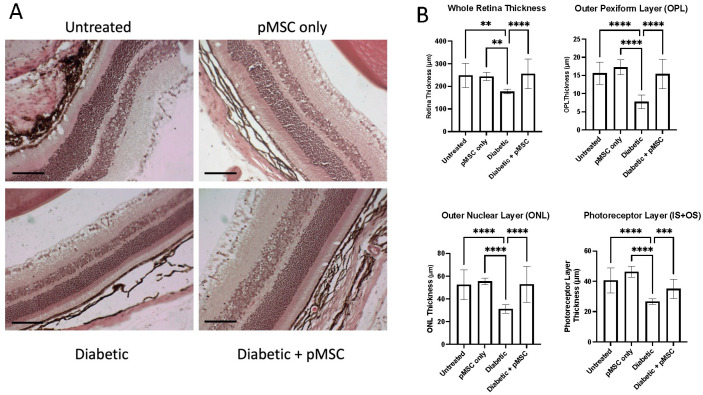
**Histological analysis of retinal tissue:** Representative images of hematoxylin and eosin (H&E) staining of retinal sections. Thickness for measurements in μm of the whole retina, outer plexiform layer (OPL), outer nuclear layer (ONL), and photoreceptor layer (IS + OS) using ImageJ analysis software. Images of H&E-stained sections were acquired at 20X magnification (**A**). Scale bar: 150 μm. Bar chart values represent the mean ± standard deviation (n = 4) (**B**). **, ***, **** Indicates significant differences (*p* < 0.01, *p* < 0.001, *p* < 0.0001).

To evaluate the effects of pMSCs on the retinal structure in diabetic mice, 4 µm thick eye sections were stained with hematoxylin and eosin (H&E) and imaged using a bright-field microscope (Figure 4A). Retinal thickness was quantified using ImageJ software, measuring the whole retina (from the ganglion cell layer (GCL) to the retinal pigment epithelium (RPE)) and sub-layers, including the outer plexiform layer (OPL), outer nuclear layer (ONL), and photoreceptor layer (IS + OS). The diabetic group exhibited a significant reduction (*p*-value < 0.05) in whole retinal thickness compared to the untreated and DBMSC-treated groups. However, pMSCs treatment in diabetic mice significantly (*p*-value < 0.05) restored whole retinal thickness (Figure 4B). Similar restorative effects were observed in the retinal sub-layers: the outer plexiform layer (OPL), outer nuclear layer (ONL), and photoreceptor layer (IS + OS) all showed significant recovery (*p*-value < 0.05) in thickness following pMSC administration (Figure 4B). These results demonstrate that pMSCs treatment effectively preserves and restores the retinal structure in STZ-induced diabetic mice, highlighting its potential to mitigate diabetic retinopathy and protect against vision-related complications in diabetes.

### 2.5. pMSCs Reduce Collagen Deposition in the Cardiac Vasculature of STZ-Induced Diabetic Mice

**Figure 5:**
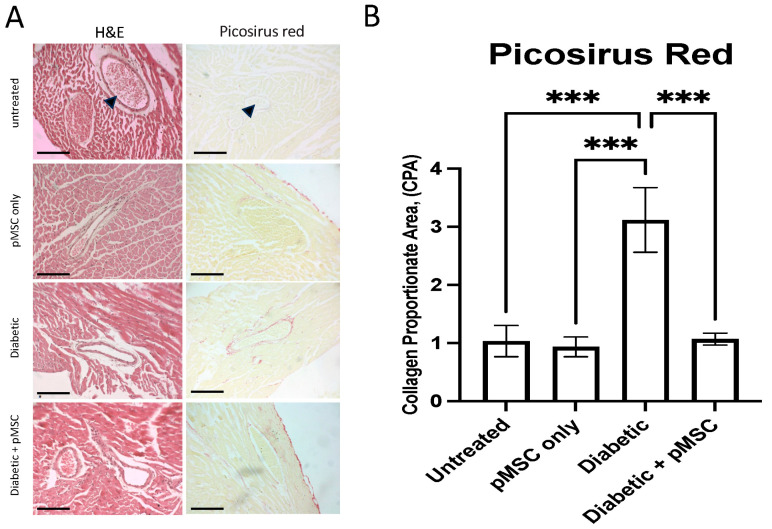
**Histological analysis of heart tissue:** Representative images of hematoxylin and eosin (H&E) staining of heart blood vessels and quantification of collagen deposition in the heart vessels are shown in panel (**A**) and (**B**), respectively. Images of H&E and Picrosirius red-stained sections were acquired at 20X magnification. Scale bar: 20 μm; Arrows indicate the blood vessels. Bar chart values represent the mean ± standard deviation (n = 4). *** Indicates significant differences (*p* < 0.001).

To assess the impact of pMSCs on the heart in diabetic mice, 4µm thick sections of cardiac blood vessels were stained with hematoxylin and eosin (H&E) and Picrosirius red. The sections were imaged using a bright-field microscope (Figure 5A), and collagen deposition in the blood vessels was quantified using ImageJ software. The results revealed a significant increase (*p*-value < 0.05) in collagen deposition in the diabetic group compared to the untreated group. However, the pMSCs treatment significantly reduced (*p*-value < 0.05) collagen deposition in the cardiac vasculature of diabetic mice compared to the untreated diabetic group (Figure 5B). These findings suggest that pMSCs effectively mitigate collagen accumulation in the cardiac blood vessels of STZ-induced diabetic mice, indicating a potential protective role against diabetes-related cardiovascular complications. This reduction in collagen deposition may help preserve vascular integrity and function in diabetic conditions.

## 3. Discussion

In this study, we aimed to evaluate the efficacy and feasibility of pMSCs in treating diabetes-induced endothelial dysfunction; we administered pMSCs intraperitoneally to STZ-induced diabetic mice. STZ is a chemical that selectively destroys pancreatic beta cells, mimicking the pathophysiology of T1D. Histological recovery was assessed using histochemistry. Although pMSCs did not normalize blood glucose levels in the STZ-induced diabetic mice, they demonstrated significant protective effects, preventing injuries to the kidney and heart. Furthermore, pMSCs maintained the structural integrity of the retina in diabetic animals. These results suggest that pMSCs may serve as a promising therapeutic tool for addressing diabetes-related endothelial dysfunction. However, further clinical studies are necessary to validate these findings and to elucidate the precise mechanisms underlying their anti-inflammatory and anti-fibrotic effects.

Diabetic nephropathy, neuropathy, retinopathy, and myocardial infarction are among the most common complications associated with both type 1 and type 2 diabetes mellitus. These complications, including kidney damage, retinal degeneration, and symptoms such as pain, numbness, tingling, and weakness in the extremities, affect approximately 50% of individuals with diabetes. In patients with chronic diabetes, prolonged hyperglycemia contributes to insufficient clearance of metabolic waste and excessive fluid retention, leading to secondary hypertension and exacerbating renal injury [18,19,20]. Current pharmacological interventions for diabetic complications primarily focus on glycemic control, blood pressure management, the regulation of glomerular hemodynamics, and the mitigation of associated comorbidities [21,22].

Animal models have a wide range of genetic, nutritional, or experimental approaches for the induction of disease and has proven to be an excellent tool to study diabetes molecular mechanisms and various forms of diabetes accompanying illnesses. A primary example of a widely, commonly used and well-established HFD-induced mouse model is described by continuous weight gain due to high fat dietary intake in combination with a genetically diabetes-susceptible mouse strain, resulting in insulin resistance mimicking the pathology of T2D, and STZ can induce T1D without the high-fat diet. Both animal models present an elevation in fasting blood glucose and an impairment in blood glucose regulation, which are frequent indicators of systemic alterations of glucose metabolism. Monitoring blood glucose and insulin levels at the normal state or after diabetes induction are easily accessible readouts. In our study, we used C57BL/6 mice, which are commonly and popularly used for inducing type 1 diabetes through low-dose administration of STZ (40 mg/kg over five days) without a high-fat diet. This model provides a reliable, consistent, and well-researched system for understanding the physiopathology of T1D and testing potential treatments, such as stem cell therapy. This model has been widely used by many researchers.

Recent preclinical and clinical studies have highlighted the therapeutic potential of adipose-derived stem cells (ASCs) in managing diabetes and its complications. Research involving animal models and human trials has demonstrated that ASC administration can effectively reduce blood glucose levels in diabetic rodents and humans. For instance, a single intravenous injection of ASCs isolated from C57BL/6 mice was shown to significantly ameliorate obesity, enhance insulin sensitivity, and restore glucose homeostasis in mice with diet-induced obesity [23,24,25,26]. These findings emphasize the promise of MSC-based therapies as a valuable approach for addressing diabetes and its associated complications.

In our previous research, we demonstrated the therapeutic potential of pMSCs in preserving endothelial cell functionality within an in vitro model of diabetes [14,23]. pMSCs were shown to counteract the adverse effects of hyperglycemia on endothelial cells by reducing apoptosis and cellular damage. Moreover, they reversed the inhibitory effects of high glucose on critical endothelial cell functions, such as proliferation, migration, survival, and angiogenesis. Additionally, pMSCs were found to attenuate endothelial cell permeability and prevent immune cell infiltration, both of which are hallmarks of endothelial dysfunction induced by elevated glucose levels [14,27]. These findings underscore the potential of pMSCs as a therapeutic strategy for addressing endothelial dysfunction in the context of diabetes.

Endothelial dysfunction is a pathological condition characterized by the impairment of endothelial cells’ biological functions. These functions include maintaining the physical and chemical barrier between blood vessels and surrounding tissues, regulating cellular adhesion, coagulation, fibrinolysis, inflammatory responses, and other essential processes involved in vascular homeostasis [23]. In diabetes, hyperglycemia is a primary driver of endothelial dysfunction, contributing to the development of severe complications such as nephropathy, retinopathy, and myocardial infarction. In this study, we aimed to evaluate the therapeutic potential of pMSCs in a mouse model of diabetes, with a focus on their ability to mitigate diabetes-associated complications.

We conducted glucose tolerance tests (GTTs) and insulin tolerance tests (ITTs) to determine the ability of pMSCs to alleviate hyperglycemia. The GTT results revealed significant glucose regulation impairments in both the diabetic group and the pMSCs-treated diabetic group compared to the untreated control group at all-time points, indicating that pMSCs were ineffective in restoring normal glucose levels. In contrast, previous studies demonstrated that ASCs were successful in regulating glucose levels in an in vivo mouse model of diabetes [23]. Similarly, the ITT results revealed no improvement in insulin sensitivity across all groups at both time points, suggesting that pMSCs had no measurable impact on whole-body insulin action. These findings align with a separate study in which ASCs also failed to enhance insulin sensitivity, further supporting the observed lack of efficacy in glucose regulation and insulin response [26].

The significant alterations in glucose levels observed in STZ-treated mice at baseline and 28 days post-STZ injection confirmed the efficacy of this well-established murine model of diabetes. The dysregulation of plasma lipid profiles in diabetes plays a critical role in the pathogenesis of diabetes-related complications [28]. This study demonstrated that no significant differences in plasma lipid profiles were observed across the experimental groups. However, a comparative analysis of baseline lipid profiles with those at 28 days post-STZ injection revealed that the observed changes were primarily associated with the standard, nutritionally balanced rodent diet and the natural physiological growth of the animals over the experimental period [29].

Fibrosis, or tissue scarring, arises from the excessive deposition of extracellular matrix (ECM) components, such as collagen, driven by chronic inflammation and repetitive tissue repair processes [13]. A reduction in collagen accumulation indicates an attenuated tissue injury and improved healing outcomes. Since pMSCs could not modulate blood glucose levels in diabetic animal models, we evaluated their therapeutic potential in addressing diabetes-associated complications, including nephropathy, retinopathy, and coronary artery disease.

In the kidney, we quantified changes in the glomerular capillary tuft area (GA) to evaluate glomerular hypertrophy. A significant increase in GA was observed across all experimental groups except the untreated control group, suggesting the presence of glomerular hypertrophy, which is a hallmark of early diabetic nephropathy. Chronic hyperglycemia leads to the expansion of glomerular endothelial and mesangial cells, a thickening of the basement membrane, and the dilatation of the capillary loops [30,31,32], which is indicative of the progression of diabetic nephropathy. In the pMSCs-treated and diabetic + pMSCs groups, the increase in tuft area may reflect vascular remodeling and cellular recruitment associated with pMSCs activity, as mesenchymal stem cells are known to secrete angiogenic and growth factors (e.g., VEGF), which can transiently influence glomerular architecture. These findings were further corroborated by a histopathological analysis using Picrosirius red staining to detect and quantify collagen deposition. The results revealed a significant increase in collagen deposition in the diabetic group. In contrast, a marked reduction in collagen deposition was observed in the pMSCs-treated diabetic group, demonstrating the protective effects of pMSCs against glomerular injury. Our results align with those of earlier studies, where it was reported that umbilical cord-derived MSCs (UC-MSCs) could effectively improve renal function, inhibit inflammation and fibrosis, and prevent the progression of diabetic nephropathy in a model of diabetes-induced chronic renal injury [33]. Studies have further demonstrated that a single intravenous administration of UC-MSCs can ameliorate glomerular abnormalities and interstitial fibrosis in a male murine model of STZ-induced diabetes, regardless of any impact on hyperglycemia. This therapeutic benefit was observed whether the UC-MSCs were administered during the early or later stages of diabetes. At both time points, the protective effects of UC-MSCs were associated with a decrease in circulating levels of transforming growth factor-beta 1 (TGF-β1) and the restoration of intra-renal autophagy [34].

To evaluate the diabetic retinopathy after STZ injection and after pMSCs injection, we quantified changes in the thickness of the entire retina and its sub-layers, including the outer plexiform layer (OPL), defined as the distance between the outer nuclear layer (ONL) and the inner nuclear layer (INL); the outer nuclear layer (ONL), defined as the distance between the outer plexiform layer (OPL) and the photoreceptor layer (PL); and the photoreceptor layer (IS + OS), defined as the distance between the outer nuclear layer (ONL) and the retinal pigment epithelium (RPE). A significant reduction in the thickness of the whole retina and its sub-layers in the diabetic group indicated structural alterations in the retinal architecture. In contrast, the pMSCs-treated diabetic group exhibited protection and restoration of the thickness of the whole retina and its sub-layers, suggesting a therapeutic effect. These findings are consistent with studies demonstrating that the intravitreal administration of ASCs can induce a cytoprotective microenvironment in the retina of diabetic mice [23]. Other studies showed that the intravitreal injection of MSC-derived small extracellular vesicles (sEVs) improved retinal function and mitigated retinal apoptosis, inflammation, and angiogenesis in STZ-induced diabetic rats. The improvement in retinal function was mediated through the inhibition of hypoxia-inducible factor-1α (HIF-1α), which led to the activation of tripartite motif 21 (TRIM21)-mediated ubiquitination and the subsequent degradation of enhancer of Zeste Homologue 2 (EZH2). This cascade ultimately resulted in the downregulation of peroxisome proliferator-activated receptor-γ coactivator-1α (PGC-1α) via EZH2-induced methylation modifications [35]. These findings further highlight the potential of MSCs as a therapeutic strategy for addressing diabetic retinopathy by targeting molecular pathways involved in diabetes-induced retinal dysfunction.

It has been demonstrated that high glucose levels upregulate TGF-β1 and phosphorylated Smad2/3 (*p*-Smad2/3) levels, leading to increased collagen fiber deposition in diabetic hearts. In contrast, bone marrow-derived mesenchymal stem cells (BMMSCs) engineered to secrete adiponectin prevented the upregulation of the TGF-β/Smad2/3 signaling pathway. This resulted in enhanced protection, suppression, and reversal of collagen deposition, reduced fibrotic area, and overall improvement in functional recovery in the hearts of diabetic rats [36]. Our study quantified collagen deposition in the heart and blood vessels to assess tissue injury and scarring. STZ-induced diabetic mice exhibited a significant increase in collagen deposition in the heart and blood vessels, indicative of glucose-induced damage. Conversely, a significant reduction in collagen deposition was observed in the pMSCs-treated diabetic group, demonstrating that pMSCs protected the heart and blood vessels from glucose-induced injury. MSCs have been shown to attenuate diabetes-induced cardiac remodeling, alleviating cardiac fibrosis and improving both systolic and diastolic function. These findings highlight MSCs as a promising therapeutic strategy for preventing diabetes-induced cardiomyopathy (DCM) [37]. However, to optimize the therapeutic potential of MSCs, it is recommended to utilize preconditioning, genetic manipulation, or advanced delivery strategies to enhance their survival, functionality, and efficacy, particularly since DM can impair the properties, quantity, and function of MSCs.

## 4. Material and Methods

### 4.1. Ethical Considerations, Collection of Placentas, and Isolation and Culture of pMSCs

This study was conducted in compliance with ethical standards and was approved on 19 January 2019, by the Institutional Review Board (IRB) and Institutional Animal Care and Use Committee (IACUC-1012-07-19) at King Abdullah International Medical Research Centre (KAIMRC) under proposal number RC17/202/R titled “Treatment and Management of Diabetes Mellitus by Human Placental Mesenchymal Stem Cells”, which ensured that all procedures adhered to established guidelines for using human samples tissues in research, prioritizing donor consent, confidentiality, and the ethical handling of biological materials. Placentas were obtained from healthy, uncomplicated pregnancies with gestational ages between 38 and 40 weeks. Donors were recruited from the Labor and Delivery Unit at King Abdul-Aziz Medical City, and informed consent was obtained from all participants prior to collection. Placentas were collected immediately after delivery and processed within 2 h to ensure the viability and integrity of the tissue for downstream applications. The use of term placentas from uncomplicated pregnancies minimized variability and ensured the quality of the isolated cells. pMSCs were isolated from the decidua basalis region of the human term placenta, as previously described [15]. The isolated cells were cultured in complete MSC-culture medium, which consisted of DMEM-F12 medium supplemented with 10% fetal bovine serum (MSC-FBS, cat#12-662-011, Life Technologies, Carlsbad, CA, USA) and antibiotics (100 μg/mL streptomycin and 100 U/mL penicillin) to prevent contamination. The cells were maintained in a controlled environment at 37 °C in a humidified incubator with 5% CO_2_ and 95% air to mimic physiological conditions. The culture medium was replaced every 2–3 days to ensure optimal growth conditions. Cell viability was assessed using a Trypan blue exclusion assay and only cultures with viability exceeding 90% were used for experiments. pMSCs were expanded in vitro and used at passage 3 to ensure consistency and avoid senescence-related changes that may occur at higher passages. Cells from twenty placentas were pooled to account for donor variability and to obtain a sufficient number of cells for the study. All clinical and experimental procedures were conducted in strict accordance with the research regulations and guidelines established by KAIMRC. This included adherence to protocols for the ethical collection, handling, and use of human tissues, as well as ensuring donor anonymity and informed consent. The study design and execution were aligned with international standards for stem cell research, emphasizing transparency, reproducibility, and ethical responsibility.

### 4.2. Induction of Diabetes in Animal Models

The animal protocol for this study was reviewed and approved by the Institutional Animal Care and Use Committee (IACUC-1012-07-19) of KAIMRC, ensuring compliance with ethical standards for the human treatment and use of animals in research. A total of twenty-four male C57BL/6 mice, aged 8 weeks, were obtained from the KAIMRC animal facility and randomly divided into two groups, control and diabetic. The mice were housed in a specific pathogen-free (SPF) environment under controlled conditions, including a 12:12 h light/dark cycle, constant temperature (22–24°C), and relative humidity (50–60%). Standard rodent chow and water were provided ad libitum, and all efforts were made to minimize stress and discomfort for the animals. Type 1 diabetes (T1D) was induced in the mice using a well-established multiple-low-dose STZ protocol, as previously described [18,19]. STZ is a diabetogenic agent that selectively targets and destroys pancreatic β-cells, mimicking the autoimmune destruction observed in human T1D. The procedure was performed as follows: (1) STZ Administration: Mice received intraperitoneal (i.p.) injections of STZ Catalog #1621, Tocris, Riyadh, Saudi Arabia, at a dose of 40 mg/kg body weight for five consecutive days. (2) Sucrose Supplementation: To counteract the initial hypoglycemic effect of STZ, mice were provided with 10% sucrose water for the five days of STZ administration. (3) Control Group: Control mice received i.p. injections of phosphate-buffered saline (PBS) for five consecutive days and were given normal drinking water. Diabetes development was monitored by measuring blood glucose levels using a glucometer. Mice were considered diabetic if their blood glucose levels exceeded 150 mg/dL on two consecutive measurements [18,19,38,39,40,41,42]. Blood glucose levels and lipid profiles were assessed on day 0 (baseline) and day 28 post-STZ injection to evaluate the progression of diabetes and metabolic changes. To ensure the reliability of the study, mice that did not develop diabetes (blood glucose levels < 150 mg/dL) or those with blood glucose levels exceeding the measurable range of the glucometer were excluded from the study. This exclusion criterion was implemented to reduce variability and potential bias in the results. On day 51 post-STZ injection, mice from both the diabetic and control groups were humanely euthanized using ketamine overdose. Tissues of interest, including the kidney, heart, and retina, were collected and dissected for subsequent histological and quantitative analyses (Supplementary Figure S1). These tissues were selected due to their susceptibility to diabetes-induced damage and their relevance to diabetic vascular complications such as nephropathy, cardiovascular disease, and retinopathy.

### 4.3. pMSCs Injections

Control and diabetic mice were randomly assigned to four experimental groups: group A (control mice, n = 6), group B (pMSC only, n = 6), group C (diabetic mice, n = 6), and group D (diabetic + pMSC, n = 6). The diabetic mice in group C were treated with an intraperitoneal injection of 0.2mL of PBS alone, while the diabetic mice in group D received an intraperitoneal injection of 1 × 10^6^ pMSCs resuspended in 0.2 mL of PBS.

### 4.4. Plasma Profile

Blood samples were collected from each animal on day 0 (baseline) and day 28 post-STZ injection to assess changes in plasma profiles following disease induction. The samples were centrifuged, and plasma was collected and stored at −80 °C for further analyses. Plasma levels of total cholesterol, triglycerides, phospholipids, free fatty acids, and glucose were measured using commercially available kits. Plasma triglycerides were quantified using an enzymatic colorimetric assay with the Triglycerides Reagent Set (cat#T531-400; Teco Diagnostics, Anaheim, CA, USA). Plasma phospholipids were measured using a phospholipid-specific enzymatic assay with Phospholipids C (cat#997-01801; Wako Chemicals, Richmond, VA, USA). Plasma cholesterol was determined using an enzymatic assay with the Cholesterol Reagent Set (cat#C509-400; Teco Diagnostics, Anaheim, CA, USA). Plasma free fatty acids were analyzed using an enzymatic assay with the HR Series NEFA-HR kit (cat#995-34791; Wako Chemicals, Richmond, VA, USA). Plasma glucose was measured using an enzymatic colorimetric assay with the Autokit Glucose kit (cat#997-03001; Wako Chemicals, Richmond, VA, USA). These analyses provided comprehensive insights into the metabolic changes associated with STZ-induced diabetes and served as a baseline for evaluating the effects of the DBMSCs treatment.

### 4.5. Glucose Tolerance Test (GTT)

All animal groups underwent a glucose tolerance test (GTT) following a previously described protocol [20]. Briefly, the animals fasted for at least 14 h with access to drinking water. Baseline body weight and glucose levels were recorded on day 0 and day 28. Blood samples (20 µL) were collected from each mouse via tail vein bleeding to measure plasma glucose levels. Mice were then injected intraperitoneally with 2 mg of glucose per gram of body weight (20% glucose solution). Blood glucose levels were measured at 15, 30, 45, 60, 90, and 120 min post-glucose administration. Animals had access to drinking water throughout the procedure. The results are presented as a line graph, and the respective areas under the curve (AUC) were calculated and plotted to assess glucose tolerance. This test provided critical insights into the animals’ ability to regulate blood glucose levels following glucose challenge, helping to evaluate the metabolic impact of STZ-induced diabetes and the potential effects of pMSCs treatment.

### 4.6. Insulin Tolerance Test (ITT)

All animal groups underwent an ITT as previously described [20]. Briefly, animals fasted for at least 2 h with access to drinking water. Baseline measurements, including body weight and glucose levels, were recorded before starting the procedure. Insulin (Novolin R, Novo Nordisk, Denmark) was administered intraperitoneally at a dose of 0.75 U/kg body weight. Blood glucose levels were measured using a One-Touch Basic glucometer (Bayer, Whippany, NJ, USA) at 15, 30, 45, 60, 90, and 120 min post-insulin administration. Animals had access to drinking water throughout the procedure. If hypoglycemia occurred, 20% glucose was administered to the animal, and it was removed from the procedure. The results are presented as a line graph, and the respective AUCs were calculated and plotted to assess insulin sensitivity. This test provided valuable insights into the animals’ ability to respond to insulin, helping to evaluate the metabolic effects of STZ-induced diabetes and the potential impact of the pMSCs treatment.

### 4.7. Histological Staining and Analysis

Organs and tissues were harvested and fixed in 4% paraformaldehyde (PFA) overnight at 4 °C. Fixed tissues were dehydrated and embedded in paraffin blocks. The dehydration process included 30 min in 70% ethanol, 60 min in 95% ethanol, 90 min in three changes of absolute ethanol, 60 min in three changes of 1:1 chloroform/xylene, 1 h in wax, and 60 min in two wax changes at 65 °C. Samples were embedded in wax blocks and left overnight at room temperature. Sections of 4 µm thickness were cut using a microtome, mounted on glass slides, and deparaffinized. Staining was performed using Hematoxylin and Eosin (H&E, Thermo Fisher, Waltham, MA, USA) and Picrosirius red (cat#ab150681, Picro Sirius Red Stain Kit, Abcam, Cambridge, UK). Stained sections were examined under a Leica DM2500 light microscope (Leica, Wetzlar, Germany).

Multiple kidney sections were stained with H&E and imaged using a Nikon Eclipse bright-field microscope at 40X resolution. ImageJ software was calibrated to convert measurements from pixels to micrometers (µm). The glomerular capillary tuft area was measured from the captured images using a screen pen. Collagen deposition was quantified and is presented as the Collagen Proportionate Area (CPA).

Multiple eye sections were stained with H&E and imaged using a Nikon Eclipse bright-field microscope at 20X magnification. ImageJ software was calibrated to convert measurements from pixels to micrometers (µm). The thickness of the whole retina, outer plexiform layer (OPL), outer nuclear layer (ONL), and photoreceptor layer (IS + OS) was quantified using a screen pen.

Multiple cardiac tissue sections were dewaxed, stained with Picrosirius red, and imaged using a Nikon Eclipse bright-field microscope (Tokyo, Japan) at 40X magnification. ImageJ software was calibrated to convert measurements from pixels to micrometers (µm). Collagen deposition was quantified and is presented as the Collagen Proportionate Area (CPA).

This comprehensive histological analysis provided detailed insights into tissue-specific damage and the protective effects of pMSCs in STZ-induced diabetic mice.

### 4.8. Statistical Analysis

Bar graphs show the data with means ± standard error (SE) from three independently executed experiments. To avoid bias, the experiments were repeated independently. Unpaired *t*-test was used for data comparison between two groups. For single data factors, two groups were compared using one-way analysis of variance (ANOVA), while data of double factors in multiple groups were compared by two-way ANOVA, and a *p* value of ≤0.05 was considered to be statistically significant.

## 5. Conclusions

We have demonstrated that while systemic administration of pMSCs does not improve insulin sensitivity in STZ-induced diabetic mice, they are effective in preventing the progression of diabetes-associated microvascular complications. These complications include nephropathy, retinopathy, and blood vessel injury in the heart. These findings underscore the potential of MSCs as a promising therapeutic strategy for preventing and reversing diabetes-induced complications. However, further research is necessary to elucidate the mechanisms underlying these protective effects before pMSCs can be translated into clinical applications.

## Data Availability

All the data generated in this study are included in this manuscript and will be available upon request.

## Supplementary Materials

The supporting information can be downloaded at: https://www.mdpi.com/article/10.3390/ijms26168057/s1.
