# Supplementary material for: Placenta-Derived Mesenchymal Stem Cells (pMSCs) Reverse Diabetes-Associated Endothelial Complications in a Preclinical Animal Model
Source: Int J Mol Sci. 2025 Aug 20;26(16):8057. doi: 10.3390/ijms26168057 (PMC12386922; doi:10.3390/ijms26168057)
Supplement: Supplementary file 1 [file ijms-26-08057-s001.zip › ijms-3642228-supplementary.pdf]

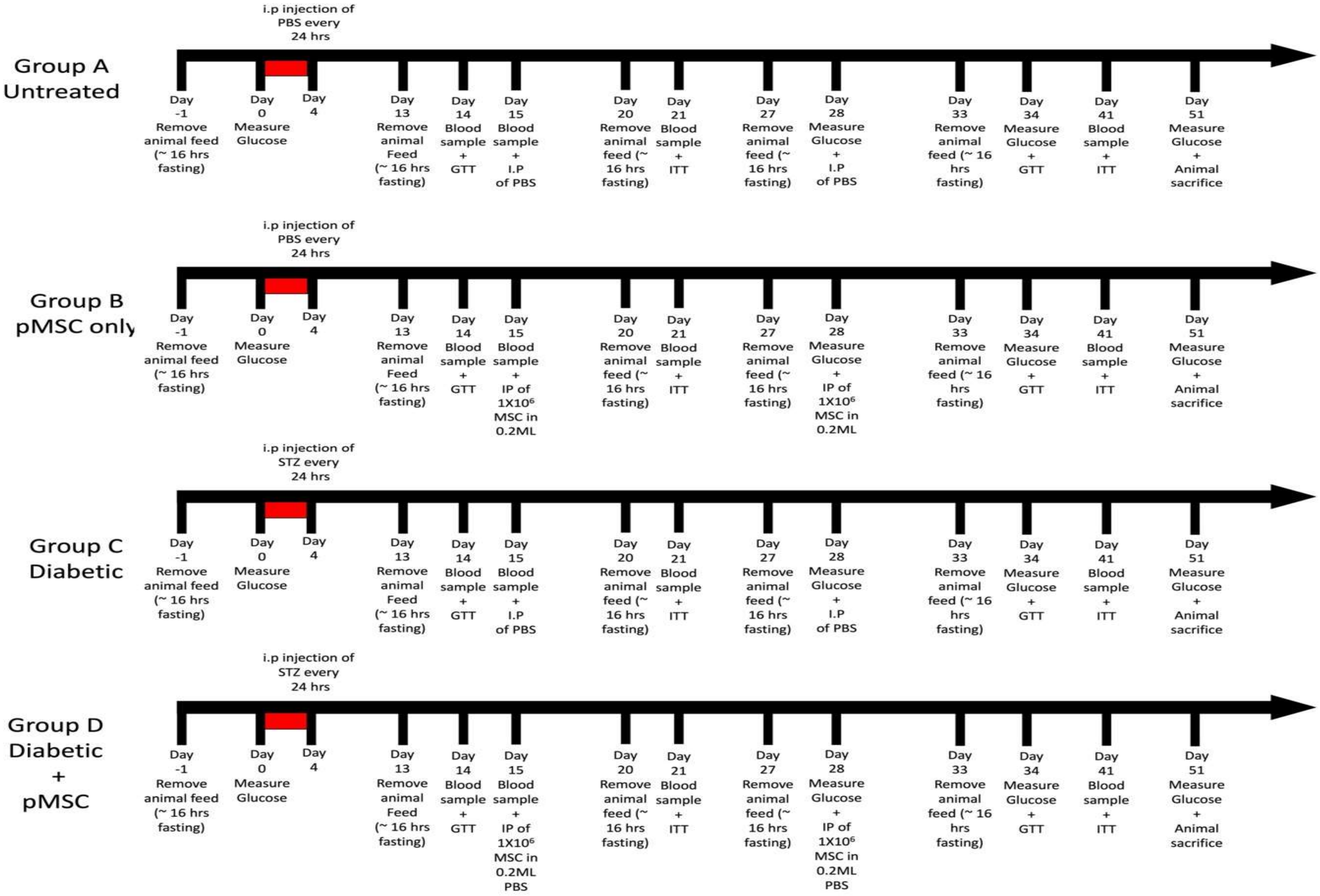

i.p injection of PBS every 24 hrs
Group A Untreated
Day -1 Remove animal feed (~ 16 hrs fasting)
Day 0 Measure Glucose
Day 4
Day 13 Remove animal Feed (~ 16 hrs fasting)
Day 14 Blood sample + GTT
Day 15 Blood sample + I.P of PBS
Day 20 Remove animal feed (~ 16 hrs fasting)
Day 21 Blood sample + ITT
Day 27 Remove animal feed (~ 16 hrs fasting)
Day 28 Measure Glucose + I.P of PBS
Day 33 Remove animal feed (~ 16 hrs fasting)
Day 34 Measure Glucose + GTT
Day 41 Blood sample + ITT
Day 51 Measure Glucose + Animal sacrifice
i.p injection of PBS every 24 hrs
Group B pMSC only
Day -1 Remove animal feed (~ 16 hrs fasting)
Day 0 Measure Glucose
Day 4
Day 13 Remove animal Feed (~ 16 hrs fasting)
Day 14 Blood sample + GTT
Day 15 Blood sample + IP of 1X10⁶ MSC in 0.2ML
Day 20 Remove animal feed (~ 16 hrs fasting)
Day 21 Blood sample + ITT
Day 27 Remove animal feed (~ 16 hrs fasting)
Day 28 Measure Glucose + IP of 1X10⁶ MSC in 0.2ML
Day 33 Remove animal feed (~ 16 hrs fasting)
Day 34 Measure Glucose + GTT
Day 41 Blood sample + ITT
Day 51 Measure Glucose + Animal sacrifice
i.p injection of STZ every 24 hrs
Group C Diabetic
Day -1 Remove animal feed (~ 16 hrs fasting)
Day 0 Measure Glucose
Day 4
Day 13 Remove animal Feed (~ 16 hrs fasting)
Day 14 Blood sample + GTT
Day 15 Blood sample + I.P of PBS
Day 20 Remove animal feed (~ 16 hrs fasting)
Day 21 Blood sample + ITT
Day 27 Remove animal feed (~ 16 hrs fasting)
Day 28 Measure Glucose + I.P of PBS
Day 33 Remove animal feed (~ 16 hrs fasting)
Day 34 Measure Glucose + GTT
Day 41 Blood sample + ITT
Day 51 Measure Glucose + Animal sacrifice
i.p injection of STZ every 24 hrs
Group D Diabetic + pMSC
Day -1 Remove animal feed (~ 16 hrs fasting)
Day 0 Measure Glucose
Day 4
Day 13 Remove animal Feed (~ 16 hrs fasting)
Day 14 Blood sample + GTT
Day 15 Blood sample + IP of 1X10⁶ MSC in 0.2ML PBS
Day 20 Remove animal feed (~ 16 hrs fasting)
Day 21 Blood sample + ITT
Day 27 Remove animal feed (~ 16 hrs fasting)
Day 28 Measure Glucose + IP of 1X10⁶ MSC in 0.2ML PBS
Day 33 Remove animal feed (~ 16 hrs fasting)
Day 34 Measure Glucose + GTT
Day 41 Blood sample + ITT
Day 51 Measure Glucose + Animal sacrifice

Supplementary Figure S1: Timeline study for Placenta-Derived Mesenchymal Stem Cell (pMSC) Treatment in Diabetic mice: The timeline study outlines the experimental procedures for pMSC treatment in a diabetic animal model. Animal feed was removed 16 hours prior to PBS or streptozotocin (STZ) administration (applied to all groups). From day 0 to day 4, PBS or STZ (40 mg/kg) was administered in multiple low doses over five days via intraperitoneal injections (PBS for groups A and B; STZ for groups C and D). On day 13, animal feed was removed from all groups. Glucose Tolerance Tests (GTT) were performed on day 14 and day 34 for all groups. On day 15 and day 28, PBS or 1 × 10^6 pMSCs were administered via intraperitoneal injections (PBS for groups A and C; pMSCs for groups B and D). On day 20, animal feed was removed from all groups. Insulin Tolerance Tests (ITT) were performed on day 21 and day 41 for all groups. On day 51, animals were sacrificed.

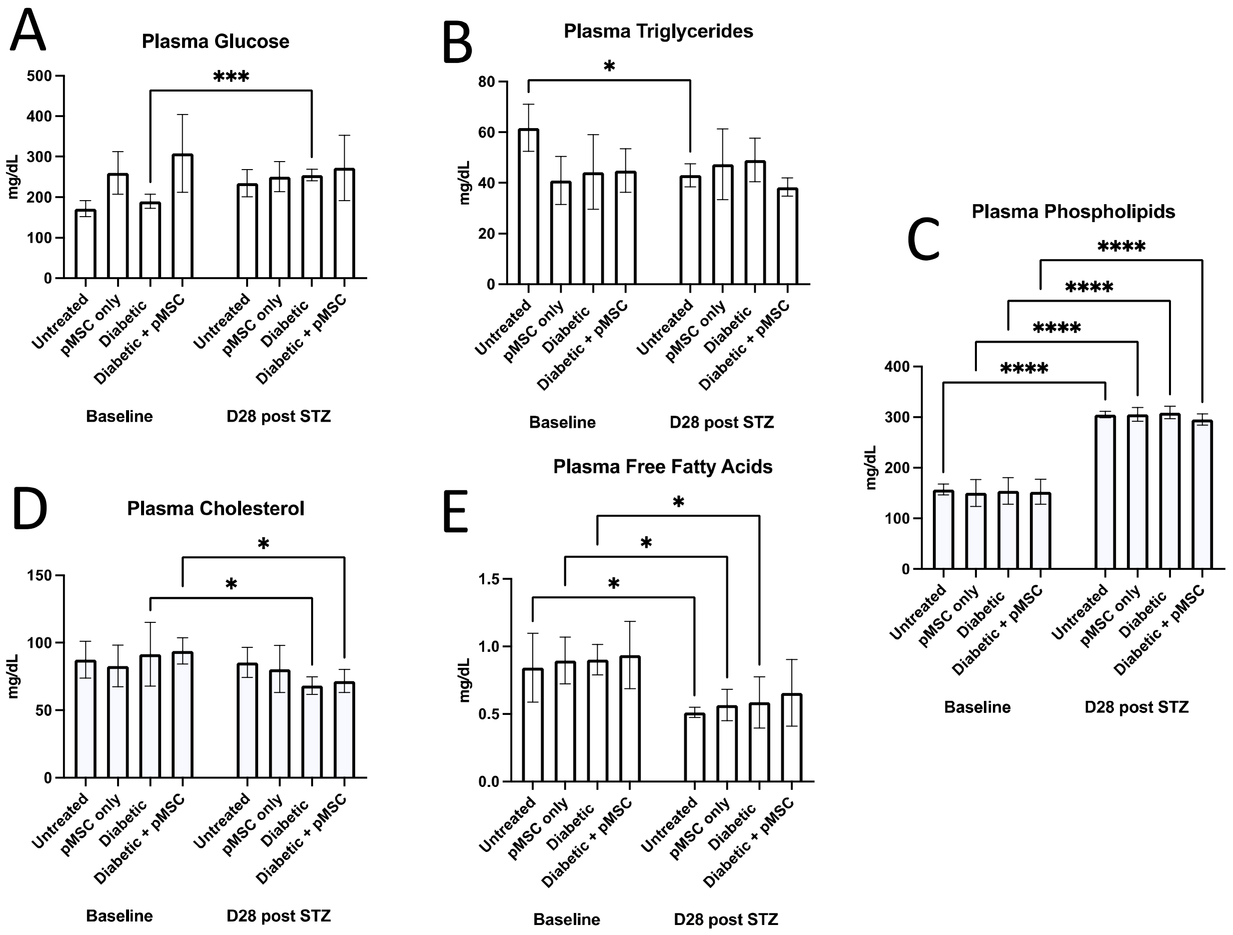

Supplementary Figure S2: Plasma Lipid Profile Analysis: Blood samples were collected on day 0 for baseline measurements and on day 28 post-STZ administration. Plasma samples were analyzed for plasma glucose (A), plasma triglycerides (B), plasma phospholipids (C), plasma cholesterol (D), and plasma free fatty acids (E). Results are presented as the mean ± standard error of the mean (SEM) (n = 6 per group; *p < 0.05 indicates significant differences).
